# Avian Polyomavirus Genome Sequences Recovered from Parrots in Captive Breeding Facilities in Poland

**DOI:** 10.1128/genomeA.00986-15

**Published:** 2015-09-24

**Authors:** Anisha Dayaram, Tomasz Piasecki, Klaudia Chrząstek, Robyn White, Laurel Julian, Katherine van Bysterveldt, Arvind Varsani

**Affiliations:** a Biomolecular Interaction Centre and School of Biological Sciences, University of Canterbury, Christchurch, New Zealand; b Department of Epizootiology with Clinic of Birds and Exotic Animals, Faculty of Veterinary Medicine, Wrocław University of Environmental and Life Sciences, Wrocław, Poland; c Department of Clinical Laboratory Sciences, Structural Biology Research Unit, University of Cape Town, Rondebosch, Cape Town, South Africa; d Department of Plant Pathology and Emerging Pathogens Institute, University of Florida, Gainesville, Florida, USA

## Abstract

Eight genomes of avian polyomaviruses (APVs) were recovered and sequenced from deceased *Psittacula eupatria*, *Psittacula krameri*, and *Melopsittacus undulatus* from various breeding facilities in Poland. Of these APV-positive samples, six had previously tested positive for beak and feather disease virus (BFDV) and/or parrot hepatitis B virus (PHBV).

## GENOME ANNOUNCEMENT

Polyomaviruses (family *Polyomaviridae*) are nonenveloped viruses with an icosahedral capsid of ~45 nm in diameter and a circular double-stranded DNA genome of ~5 kb. The bidirectionally transcribed circular genome encodes three structural proteins, VP1, VP2, and VP3, on one strand, and transforming nonstructural protein genes, the large and small T antigens, on the complementary strand. Numerous polyomaviruses have been identified and infect a wide range of vertebrates. Currently, all known polyomaviruses can be assigned to three genera: *Orthopolyomavirus* and *Wukipolyomavirus*, which encompass polyomaviruses of mammalian origin, and *Avipolyomavirus*, which infects birds. Documented avipolomaviruses include Adélie penguin polyomavirus, butcherbird polyomavirus, canary polyomavirus, crow polyomavirus, finch polyomavirus, goose hemorrhagic polyomavirus, and the avian polyomaviruses (APVs) (formerly known as budgerigar fledgling disease virus), which infect various parrot species (1–6). APV infections in parrots can cause clinical symptoms in some species (7), inducing chronic disease of the skin and feathers, and frequently, coinfection with beak and feather disease virus (BFDV) (8, 9).

In order to identify APVs circulating in various breeding facilities in Poland, total DNA was extracted from liver samples collected between 2007 and 2011 from 26 deceased parrots (*Melopsittacus undulatus*, *n* = 6; *Platycercus elegans*, *n* = 2; *Psittacula eupatria*, *n* = 1; *Psittacula krameri*, *n* = 15; *Psittacus erithacus*, *n* = 1; and *Trichoglossus haematodus*, *n* = 1), as previously described (10–12). Total DNA was enriched by rolling circle amplification using the illustra TempliPhi amplification kit (GE Healthcare, USA), and the concatenated DNA was digested separately with BamH1 and Xmn1 restriction enzymes. The resulting ~5-kb fragments were gel purified and cloned into pJET1.2 (Thermo Fisher) for XmnI-restricted products and pGEM 3Zf(+) (Promega Biotech, USA) for BamHI-restricted products. The cloned products were Sanger sequenced by primer walking at Macrogen, Inc. (South Korea), and the sequence contigs were assembled using the DNA Baser sequence assembler version 4.16 (Heracle BioSoft SRL, Romania). Of the 26 samples tested, eight birds from three species were found to be positive for APV. They were *P. eupatria* (*n* = 1; PL830), *P. krameri* (*n* = 4; PL904, PL1025, PL1220, and PL1233), and *M. undulatus* (*n* = 3; PL1067, PL1068, and PL1233). The viral genomes were fully sequenced and the analyzed genome-wide identity calculated using SDT version 1.2 (13). The genomes share >99.6% identity, while the overall diversity of known APVs (calculated by the inclusion of the 14 genomes available in GenBank) is 0.8%. It is worth noting that six of the eight APV-infected liver samples reported here also contained BFDV (10) and/or parrot hepatitis B virus (PHBV) (11). Two samples, *P. eupatria* (PL830) and *P. krameri* (PL1233), were coinfected with both BFDV and PHBV, while PHBV alone had been identified in two additional APV-infected *P. krameri* strains (PL904 and PL1220) (11). BFDV was also previously identified in two *M. undulatus* strains (PL1067 and PL1068) (10). Neither BFDV nor PHBV was detected in PL1025 (*P. krameri*) or PL1225 (*M. undulatus*).

This short communication provides the genome sequences of eight new APVs from three captive parrot species and shows the relatively low diversity of the known APV pool, which so far comprises genome sequences from China, Germany, Japan, and Poland, which have been recovered from various parrot species.

### Nucleotide sequence accession numbers.

The complete genome sequences have been deposited at GenBank under the accession numbers KT203762 to KT203769.
